# When Blue Turns the Green Off: Implications of Methylene Blue Interference in Indocyanine Green Near-Infrared Fluorescence Imaging

**DOI:** 10.3390/ani16060983

**Published:** 2026-03-21

**Authors:** Elisa Maria Gariboldi, Luigi Auletta, Roberta Ferrari, Alessandra Ubiali, Damiano Stefanello

**Affiliations:** Dipartimento di Medicina Veterinaria e Scienze Animali, Università degli Studi di Milano, 26900 Lodi, Italy; luigi.auletta@unimi.it (L.A.); roberta.ferrari@unimi.it (R.F.); alessandra.ubiali@unimi.it (A.U.); damiano.stefanello@unimi.it (D.S.)

**Keywords:** quenching, lymphography, sentinel lymph node, fluorescence intensity quantification

## Abstract

Sentinel lymph node mapping is increasingly used in canine and feline oncology and often involves the combined use of visible dyes and fluorescent tracers. However, the effect of methylene blue on the fluorescence of indocyanine green during near-infrared imaging remains unclear. This explorative study aimed to quantitatively and qualitatively assess potential fluorescence quenching in solutions of methylene blue–indocyanine green at different ratios in three near-infrared imaging modalities (overlay, color map, contrast). Four solutions were prepared: 100%/0%, 75%/25%, 50%/50%, and 25%/75% indocyanine green/methylene blue. The fluorescence intensity of the four solutions was quantitatively measured in vitro using near-infrared imaging. Subsequently, four lymphographies, one for each solution, were performed from the metatarsal region of feline cadavers. Observers with varying levels of experience evaluated lymphographic images. Methylene blue caused a concentration-dependent reduction in fluorescence both at the quantitative evaluation and qualitative lymphography interpretation. Despite this reduction, fluorescence remained sufficient in cadavers for accurate identification of lymph nodes, and observer experience did not significantly affect interpretation, except for the color map mode. Because methylene blue-dominant solutions showed a greater quenching effect on indocyanine green fluorescence, clinicians should favor indocyanine green-dominant mixtures. This approach may preserve fluorescence performance, maintaining the surgical guidance benefits of methylene blue. Future confirmatory studies should include a substantially larger number of specimens to allow appropriate statistical comparisons and to better account for inter-individual variability.

## Simple Summary

The accurate identification of the lymph node that drains a tumor (sentinel lymph node) is essential for cancer staging in pets. To this end, dyes are injected to identify lymph nodes and guide surgeons in their removal. Two commonly used tracers are methylene blue and indocyanine green. Indocyanine green emits fluorescence that can be detected with near-infrared cameras, helping surgeons visualize lymphatic vessels and lymph nodes. These agents are sometimes used together, but it is unclear whether methylene blue could reduce the fluorescence intensity of indocyanine green, which could make lymph nodes harder to detect with lymphography. This exploratory study investigated whether methylene blue affects the fluorescence of indocyanine green in sentinel lymph node mapping. This study quantified fluorescence intensity in different mixtures of these two tracers in vitro and then qualitatively interpreted lymphography in cat cadavers. Observers with varying levels of experience evaluated fluorescence intensity and the lymphographic images. The results showed that higher concentrations of methylene blue reduced fluorescence, but the signal remained sufficient to correctly interpret the lymphography. These findings indicate that combining methylene blue with indocyanine green may be effective for sentinel node mapping in pets. Future confirmatory studies should include a substantially larger number of specimens to allow appropriate statistical comparisons and to better account for inter-individual variability.

## 1. Introduction

Indocyanine green (ICG) and methylene blue (MB) are among the most commonly used dye-based agents, administered either alone or in combination, in both canine and feline patients for sentinel lymph node (SLN) mapping and extirpation [1,2,3], as well as in humans [4,5,6,7]. They show distinct properties and visualization mechanisms in clinical applications. MB is a visible dye that in many cases can only be identified after surgical exposure of the lymphatic vessels or lymph node after cutaneous and subcutaneous tissues dissection [8,9,10]. For this reason, MB is generally associated with other tracers that can map the lymphatic network and guide tissue dissection to reach the SLN, such as ICG [1,2,11,12].

Meanwhile, ICG is a fluorophore that emits near-infrared fluorescence (NIRF), which can be captured in real-time by specialized NIRF imaging systems. The ICG fluorescence identification may be influenced by several factors, including the environmental conditions, operating room lighting, and fluorophore concentration [13,14,15,16,17]. In addition, ICG fluorescence may undergo “quenching” phenomena, a reduction in fluorescence intensity that can impair its performance. Two main types of quenching effects have been described: self-quenching, which occurs when high fluorophore concentrations lead to molecular aggregation and reduced emission efficiency [6,14], and intermolecular quenching, which results from interactions between fluorophores and other molecules that dissipate the excitation energy [15]. Previous studies have mainly focused on ICG self-quenching, which occurs at high fluorophore concentrations and is reduced by dilution [6,14]. Conversely, data regarding a possible intermolecular quenching effect, e.g., by MB on ICG fluorescence, is lacking and needs to be explored. Indeed, although MB and ICG seem to be routinely used together in daily clinical practice [1,2,11,12], there is still limited evidence regarding intermolecular quenching when mixed before injection. To the best of the authors’ knowledge, only one clinical study in cats has reported a reduction in ICG fluorescence when administered in combination with MB [18]. In that study, when ICG and MB were combined in solution, the fluorescence intensity within the SLN was lower compared to ICG alone, suggesting that ICG alone should be preferred over an ICG–MB combination [18]. However, the significance of this reduced fluorescence intensity remains unclear in a clinical setting. Furthermore, to the best of the authors’ knowledge, to date, no study has evaluated the optimal ICG/MB ratio for achieving high-quality fluorescence visualization while maintaining the benefits of MB visualization during tissue dissection in SLN removal.

In this context, the present study aimed to explore the potential occurrence of an intermolecular quenching effect on the fluorescence emitted by ICG when mixed with MB at different dilution ratios. Specifically, the study wants to determine whether such an effect may quantitatively influence the measurement of fluorescence intensity (Part 1: in vitro study) and to conduct an initial investigation into whether it may qualitatively interfere with the interpretation of lymphographic images (Part 2: cadaveric study in feline model). Additionally, since the near-infrared fluorescence imaging system used in this study provides three observation modalities (overlay, color map, and contrast), a secondary objective was to compare these modalities in terms of fluorescence signal visualization and interpretation.

## 2. Materials and Methods

All described procedures were conducted at the Veterinary Teaching Hospital (VTH) of the University of Milan, Lodi, Italy. For Part 2 of the study, written consent for the scientific use of the cadavers for educational and research purposes was obtained from the owners of cats or from the public veterinary officer in the case of unowned free-roaming cats.

This explorative study evaluated the fluorescence of 4 solutions: an ICG-only solution (100% ICG) and 3 ICG-MB solutions with different proportions:

- ICG-dominant (75% ICG/25% MB);
- ICG-MB equal (50% ICG/50% MB);
- MB-dominant (25% ICG/75% MB).

The experiments consisted of two parts:

- Part 1: In vitro testing for quantification of fluorescence intensity of the 4 solutions;
- Part 2: Qualitative interpretation of cadaveric lymphographic images from feline cadavers using the 4 solutions.

### 2.1. Dyes and NIRF Technology

For solution preparation, ICG (Verdye, Diagnostic Green, Garrycastle, Ireland) was reconstituted with sterile water for injectable preparations to obtain a stock solution at a concentration of 1.25 mg/mL. The MB solution was used at the concentration sold by the manufacturer of 5 mg/mL, without further dilution (S.A.L.F. S.p.A., Cenate Sotto, Bergamo, Italy).

Fluorescence was assessed using the NIRF camera SPY-PHI QP% system (PINPOINT system, Stryker/Novadaq, model PC9001; Stryker; MIDA, Tecnologia Medica S.p.a., Milan, Italy) with a Portable Handheld Imager (SPY-PHI). The system operates with a laser excitation peak at 805 nm and detects fluorescence within an emission band of approximately 825–850 nm. The SPY-PHI QP% system (Stryker) allows three visualization modalities for fluorescence, defined as “overlay”, “color map”, and “contrast” mode. Moreover, the technology can also be used in “white light” mode (no fluorescence). The system includes QP% software, which enables real-time quantification of fluorescence intensity. With this technology, the point of maximum fluorescence must be defined as QP% 100%, after which the QP% of various regions of interest (ROIs) can be evaluated.

To minimize interference from ambient light, all images were obtained in a darkened room with both natural and artificial lights excluded. In both Part 1 and Part 2, the camera–sample distance was kept constant at 15 cm and images were acquired at a 90° angle relative to the sample surface to avoid variability in NIRF signal detection.

All images acquired using the NIRF system were saved and used for both Phase 1 and Phase 2 of the study and were visualized using the built-in high-definition display mode included in the SPY-PHI (Stryker) system.

### 2.2. Part 1: Quantification of Fluorescence Intensity In Vitro Testing

Since the in vitro tests lacked tissue proteins to which ICG could bind, an albumin solution (Albumina Grifols^®^ 200 g/L, Istituto Grifols S.A., Frankfurt, Germany) had to be added to enable fluorescence emission [19]. The albumin solution had an initial concentration of 200 mg/mL and was diluted in 0.9% NaCl solution to achieve a final concentration of 35 mg/mL (ALB), which is comparable to the physiologic reference range of feline and canine serum albumin (25–40 mg/mL). The 4 solutions used in Part 1 had a total volume of 1.2 mL and were prepared as follows:

- ICG-only (ICG concentration 0.833 mg/mL): ICG (0.8 mL) + ALB (0.4 mL);
- ICG-dominant (ICG concentration 0.625 mg/mL): ICG (0.6 mL) + MB (0.2 mL) + ALB (0.4 mL);
- ICG-MB equally mixed (ICG concentration 0.416 mg/mL): ICG (0.4 mL) + MB (0.4 mL) + ALB (0.4 mL);
- MB-dominant (ICG concentration 0.208 mg/mL): ICG (0.2 mL) + MB (0.6 mL) + ALB (0.4 mL).

**Figure 1:**
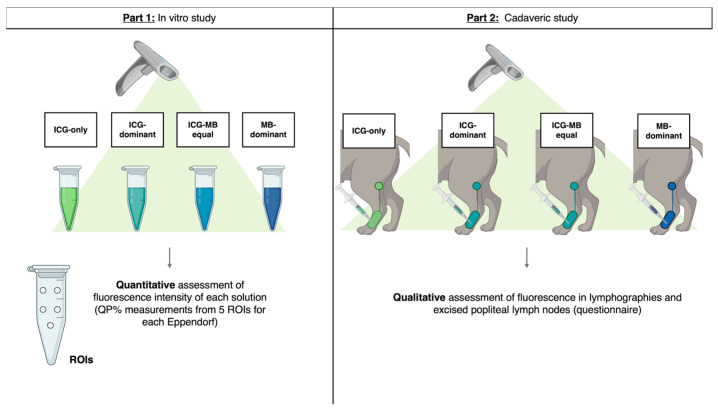
Schematic representation of the two parts of the study. On the left, Part 1 (in vitro study), showing the 4 solutions used in both Part 1 and 2, reconstituted in Eppendorf tubes for the quantitative evaluation. On the right, Part 2 (cadaveric lymphography), displaying the cutaneous area and the lymph node selected for the lymphography evaluation.

The 4 solutions were deposited in 4 distinct Eppendorf tubes. The Eppendorf tubes were placed on a work surface (table) covered with a surgical drape of the same type as those routinely used in surgery and employed in Part 2 of the study (cadaveric phase). The NIRF images of the 4 solutions were acquired in all three NIRF modalities. Then, the fluorescence intensity of each solution was measured. The Eppendorf tube with ICG-only was used as referral point of maximum fluorescence to define the reference QP% 100%. The fluorescence intensity of the other solutions was directly calculated with the QP% system using the SPY-PHI. For each Eppendorf tube, five different, reproducible ROIs (as reported in Figure 1) were selected and measured for the QP% in each of the three modalities, and data were recorded for subsequent analysis.

### 2.3. Part 2: Qualitative Interpretation of Cadaveric Lymphography

The 4 solutions prepared for Part 2 were prepared using the same proportions of ICG and MB as described for Part 1, but without the addition of albumin (ALB) for a total volume of 0.8 mL for each solution.

Adult feline cadavers were included and underwent NIRF lymphography according to a previously described protocol [20], each of which was performed with one of the 4 dye solutions. Cadavers derived from cats referred to VTH and euthanized or deceased due to causes unrelated to the study. Only cadavers of cats that had died within 24 h prior to the procedure and had not been previously frozen were used; specimens showing severe post-mortem alterations or macroscopic hind limb abnormalities were excluded.

To ensure reproducibility, the dorsal metatarsal region was selected for the lymphography, since it exhibits a predictable lymphatic drainage toward the popliteal lymphocentrum [20]. Drainage toward a different lymphocentrum or interrupted lymphatic pathways would have led to the exclusion of the lymphography from the study. A detailed description of cadaver handling, anatomical landmarks, tracer injection, and lymphography procedures is provided in Appendix A. Four hind limbs were used for cadaveric lymphography, with one limb assigned to each of the 4 solutions. For each solution, a single suitable lymphography was evaluated. Images were acquired in overlay, color map, and contrast modes. All popliteal lymph nodes identified during the four lymphographies were excised and individually evaluated across all NIRF modalities.

A total of 24 NIRF images with all NIRF modalities (12 from the 4 lymphographies and 12 from the 4 excised popliteal lymph nodes) were collected. A questionnaire was developed to qualitatively assess fluorescence in the 24 NIRF images. The questionnaire was used to evaluate the fluorescence of lymphographic images and excised lymph nodes across the different dye solutions and the various NIRF modalities. Images were presented to the observers in random order, without any indication about the ICG concentration of the injected solutions, so that the observers were completely blinded to the solution used.

Respondents were assigned to three groups of evaluators (*n* = 4 per group) based on experience in interpreting NIRF images:

- High-experience group: Four faculty members of the Small Animal Soft Tissue Surgery and Oncology Service at the VTH with 4 years of daily clinical use of NIRF technology.
- Moderate-experience group: Four volunteer veterinary students (tenth semester) attending the Small Animal Soft Tissue Surgery and Oncology Service at the VTH, with at least one year of daily involvement in NIRF-based clinical procedures.
- Low-experience group: Four veterinary students attending their sixth to eighth semesters, without prior exposure to NIRF technology or knowledge of SLN or fluorescence-guided surgery. Participants were selected from volunteer students rotating through teaching activities at the VTH between March and May 2025.

The assessment was based on visual interpretation of fluorescence imaging using a questionnaire. For each image, respondents were asked two questions:

- Question 1: Recognizability of fluorescence in lymphographies and lymph nodes: “Is the fluorescence of the lymphography/lymph node recognizable?” (yes/no)
- Question 2: Perceived score of fluorescence intensity in lymphographies and lymph nodes: “How fluorescent does it appear to you, on a scale from 1 (not fluorescent) to 5 (highly fluorescent)?”

Phase 1 and Phase 2 of the study are summarized in Figure 1.

### 2.4. Statistical Analysis

Statistical analysis was performed using IBM SPSS Statistics for macOS (v.30.0; IBM Corp., Armonk, NY, USA) and GraphPad Prism for macOS (version 10.1.1; GraphPad Software, San Diego, CA, USA).

Quantitative fluorescence intensity data from the in vitro analysis were tested for normality within each solution and each NIRF modality with the Shapiro–Wilk *W* test. For the ICG-only and -dominant in all three NIRF modalities, the ICG-MB equal in overlay and color map, and the MB-dominant in color map and contrast modes, solution fluorescence intensities are expressed as mean ± standard deviation, whereas for the ICG-MB equal in contrast mode and the MB-dominant in overlay, they are expressed as median (range), according to the data distribution. The values of the five measurements per solution have been compared between NIRF modalities and the 4 solutions via an ordinary two-way ANOVA, with Bonferroni correction for multiple comparisons. The effects of the four ICG solutions and of the NIRF modalities are reported. Afterwards, all fluorescence intensity values were plotted against the MB concentration in the 4 solutions (i.e., 0.0, 0.25, 0.50, and 0.75) and their correlation was assessed using Spearman’s rank correlation coefficient (r_s_).

For the qualitative assessment of lymphography and excised popliteal lymph nodes in cadavers, questionnaire scores were treated as non-parametric variables. The proportion of observers recognizing fluorescence was calculated for each tracer solution, visualization mode, and observer expertise level. Differences in fluorescence recognition rates among tracer solutions were evaluated using the chi-square test. The standardized residuals (z_i_) and the relative Bonferroni adjusted *p*-values (pB) have been calculated to evaluate which ICG solution would have been significantly associated with fluorescence recognition [21]. Differences in fluorescence scores between observer expertise groups (high, moderate, and low experience) for each of the 4 solutions within each NIRF visualization mode were assessed using the Kruskal–Wallis test; post hoc, differences between each group were tested with Dunn’s multiple comparisons test.

Statistical significance was set at *p* < 0.05.

## 3. Results

### 3.1. Part 1: Quantification of Fluorescence Intensity In Vitro Testing

**Figure 2:**
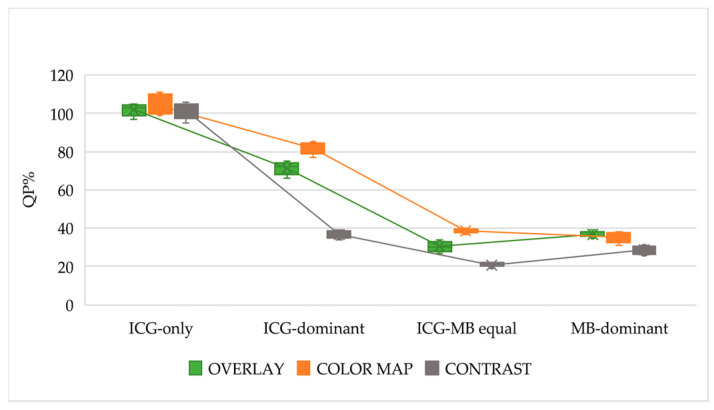
Graphic representation of the median (range) fluorescence intensity for the 4 solutions in the three NIRF modalities.

**Figure A1:**
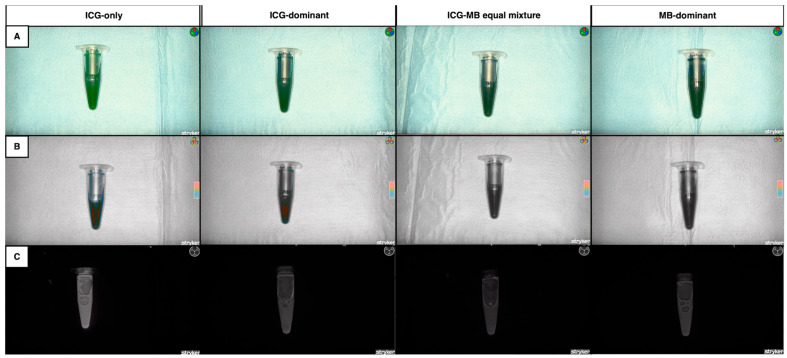
The visualization of the 4 ICG-MB solutions in the in vitro study. (**A**): Overlay mode; (**B**): color map mode; (**C**): contrast mode.

In the in vitro analysis, NIRF registered an overall progressive and significant attenuation of fluorescence intensity accompanying the increase in MB concentration in the solutions (*p* < 0.0001; Figure 2; Table A1). The NIRF modality analysis showed significant differences (*p* < 0.0001) within the ICG-dominant, ICG-MB equal, and MB-dominant solutions (Appendix A Table A1). A strong negative correlation between MB concentration and fluorescence intensity (r_s_ = −0.801, *p* < 0.0001) was detected. The NIRF images of the four solutions in the in vitro study are reported in Appendix A (Figure A1).

### 3.2. Part 2: Qualitative Interpretation of Cadaveric Lymphography Based on a Questionnaire

**Figure A2:**
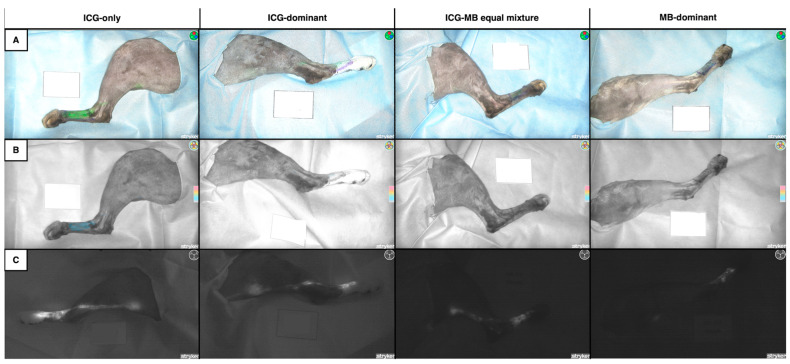
The 4 lymphographies by the 4 ICG-MB solutions in the cadaveric study. (**A**): Overlay mode; (**B**): color map mode; (**C**): contrast mode.

**Figure A3:**
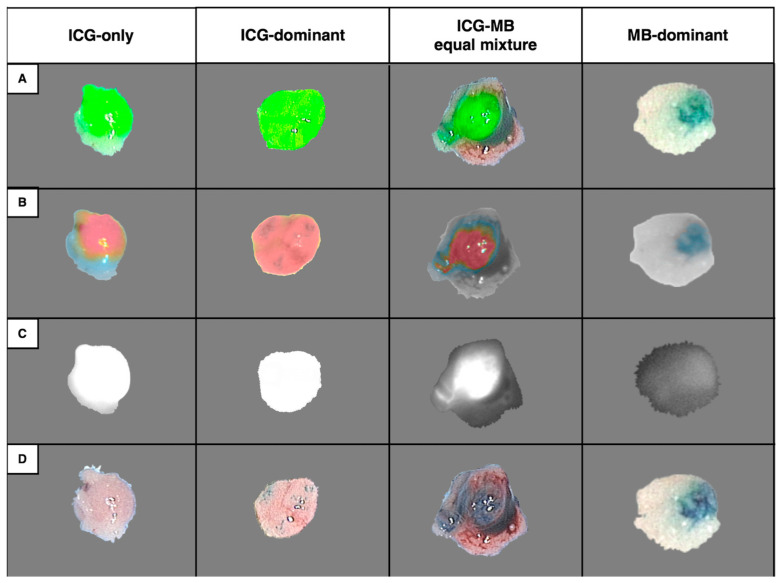
The 4 excised popliteal lymph nodes with 4 ICG-MB solutions in the cadaveric study. Rows: (**A**): Overlay mode; (**B**): color map mode; (**C**): contrast mode; (**D**): white light mode.

To obtain the four feline hind limbs suitable for the lymphographies, a total of four cats (eight hind limbs) were selected. Two hind limbs were excluded due to the presence of cutaneous lesions; one hind limb was excluded because the lymphatic drainage migrated to the inguinal lymphocentrum rather than the popliteal one, and one hind limb lymphography displayed an interrupted lymphatic pathway; i.e., the drainage stopped before the popliteal lymphocentrum without reaching it. The four hind limbs included showed successful lymphatic migration to the popliteal lymph node. The NIRF images of Part 2 are reported in Appendix A (Figure A2 and Figure A3).

#### 3.2.1. Questionnaire—Question 1: Recognizability of Fluorescence in Lymphographies and Lymph Nodes

Evaluating the lymphographies, in overlay mode, the proportion of participants recognizing fluorescence was significantly different between the four solutions (χ^2^ = 22.0, df = 3, *p* < 0.0001), with the highest proportion observed for the ICG-only solution (100%, z_i_ = +2.45, pB = 0.11) and the lowest for the MB-dominant mixture (8%, z_i_ = −2.04, pB = 0.33). In color map mode, the proportion of participants recognizing fluorescence was significantly different between the four solutions (χ^2^ = 39.9, df = 3, *p* < 0.001), with the significantly highest proportion observed for the ICG-only solution (100%, z_i_ = +4.54, pB = 0.00004). The contrast mode achieved uniform fluorescence recognition across all solutions (100%), regardless of tracer composition (Table 1). For excised popliteal lymph nodes, fluorescence recognition was 100% across all solutions in color map and contrast modes (Table 1). In overlay mode, there was a significant difference in the proportion of observers identifying fluorescence (χ^2^ = 24.6, df = 3, *p* < 0.001), with the MB-dominant solution displaying a significantly reduced recognition rate (58%, z_i_ = +3.97, pB = 0.0006).

#### 3.2.2. Questionnaire—Question 2: Perceived Score of Fluorescence Intensity in Lymphographies and Lymph Nodes

**Figure 3:**
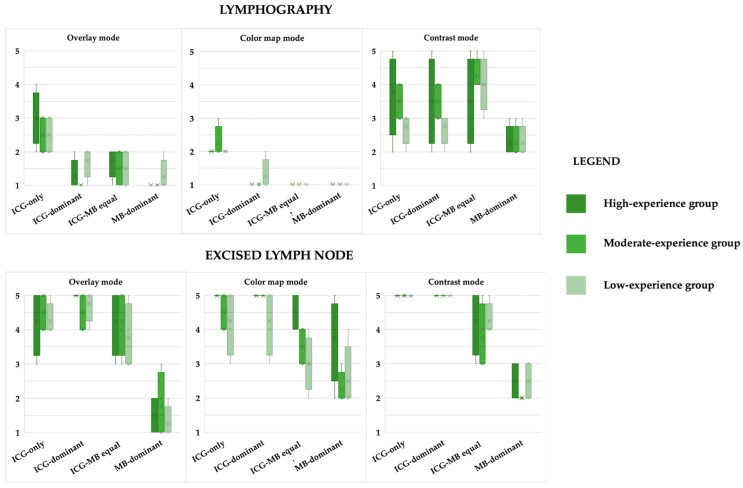
Subjective scores of fluorescence intensity collected from the questionnaire for the 4 ICG-MB solutions in lymphography and excised lymph nodes.

No significant differences were observed between participants with high, moderate, or low levels of experience, for any of the four solutions and the NIRF modes for the evaluation of the lymphographies. No significant differences were observed between participants with high, moderate, or low levels of experience, for any of the four solutions and the NIRF modes for the lymph nodes, except for the ICG-MB equal solution in color map mode (*p* = 0.049). In particular, the group with high experience showed significantly higher median scores compared to the low-experience group (*p* = 0.011). The results are summarized in Table 2 and Figure 3 and specific *p*-value are summarized in Table A2.

Moreover, the lymphographies, were significantly different between the four solutions for all visualization modes (overlay: *p* < 0.0001; color map: *p* < 0.0001; contrast: *p* = 0.0003) (Figure 3). In overlay and color map modes, the ICG-only solution consistently achieved the highest median scores compared to all other solutions (*p* < 0.0001, for all). Moreover, only in the overlay mode, the MB-dominant formulation showed a median score lower than the ICG-MB equal solution (*p* = 0.019). In contrast mode, the MB-dominant formulation showed a median score lower than all other solutions (ICG-only *p* = 0.002, ICG-dominant *p* = 0.004, ICG-MB equal *p* < 0.0001), and the ICG-MB equal solution showed a higher median score than the ICG-dominant solution (*p* = 0.047).

For the isolated lymph nodes, fluorescence scores were significantly different between the four solutions for all visualization modes (overlay: *p* < 0.0001; color map: *p* < 0.0001; contrast: *p* < 0.0001) (Figure 3). In the overlay mode, the MB-dominant formulation showed a median score lower than all other solutions (*p* < 0.0001, for all), and the ICG-dominant solution showed a higher median score than the ICG-MB equal solution (*p* = 0.022). In the color map mode, the MB-dominant formulation showed a median score lower than all other solutions (ICG only and ICG dominant *p* < 0.0001, ICG-MB equal *p* = 0.020), and the ICG-MB equal solution showed a median score lower than both the ICG-only and ICG-dominant solutions (*p* = 0.011 and *p* = 0.003, respectively). In the contrast mode, the MB-dominant formulation showed a median score lower than all other solutions (*p* < 0.0001, for all), and the ICG-MB equal solution showed a median score lower than both the ICG-only and ICG-dominant solutions (*p* < 0.0001, both). The results of Question 2 for lymphography and lymph nodes are summarized in Figure 3.

## 4. Discussion

The present study explored the intermolecular quenching effect exerted by MB on ICG fluorescence when mixed in solutions for SLN mapping. The fluorescence attenuation was observed both quantitatively, through in vitro analyses (Part 1), and qualitatively, through the interpretation of feline cadaveric lymphographic images by three groups of people with different experience levels in the use of ICG in a clinical setting (Part 2).

Our results highlight that the ICG-only solution is unquestionably associated with the highest fluorescence intensity in comparison with ICG-MB solutions (Figure 2). On the other hand, increasing the proportion of MB in the solutions leads to a reduction in fluorescence intensity in the in vitro analyses and to a decreased ability to interpret fluorescence in lymphographic images in the feline cadaveric study. Moreover, ICG-only was the only solution that never failed to achieve fluorescence detection in both lymphographies and the ex vivo lymph nodes, and it consistently received the highest scores from observers across all levels of expertise (Table 1; Figure 3). In agreement with previous reports by Lu et al. (2023) [18], our findings indicate that ICG-only solutions provide stronger and more clearly detectable fluorescence signals, which can be more easily appreciated without the need for a specific learning curve.

Nevertheless, it is worth noting that, although MB is not generally visible through tissues unless directly exposed, it enhances the visualization of lymph nodes within the surrounding tissues, thereby facilitating their identification and guiding the surgeon during dissection for SLN removal, as is well established in both the veterinary and human literature [1,2,4,5,6,7,8,10]. Given that our results clearly indicate that the ICG-only approach represents the best-performing solution for fluorescent lymphography, the ICG-dominant configuration emerged as the most effective option compared to the other ICG–MB combinations. Considering the surgical aid provided by MB [1,2,4,5,6,7,8,10], future clinical studies are warranted to confirm the advantage of the ICG-dominant solution in an in vivo clinical setting.

Getting deeper into the quantitative fluorescence intensity (Part 1: in vitro study), a reduction in fluorescence intensity is observed from the ICG-only solution to the first ICG-MB solution, in which ICG remains the dominant component; fluorescence intensity decreases even more markedly in the ICG–MB equal solution. A further decrease in fluorescence intensity was expected to be observed in the MB-dominant solution, but instead, no further reduction in fluorescence intensity was observed (Figure 2). It might be hypothesized that, at this specific dilution, the intermolecular quenching effect exerted by MB on ICG fluorescence is outweighed by the dilution-related attenuation of self-quenching. Indeed, the larger volume of MB increases the dilution of ICG, thereby reducing ICG self-quenching. Further investigations in biochemistry and optical physics are required to confirm this mechanism.

As an explorative study conducted in a cadaveric model, in the qualitative fluorescence analysis in the interpretation of lymphography images (Part 2: cadaveric study), a significant difference among the various dilutions was registered, supporting the finding that increasing MB concentration reduces the operator’s ability to recognize ICG fluorescence during lymphography. However, a further distinction must be made among the different NIRF modalities. Indeed, a greater difficulty in visualizing fluorescence was observed in the “overlay” and “color map” modes. Conversely, the “contrast” mode never showed failures in fluorescence recognition, not even in the MB-dominant solution. This could suggest that the “contrast” mode is the most suitable setting during mapping for a better and constant identification of fluorescence of lymphatic drainage and superficial lymphocenters.

Considering the two parts of the study (Part 1 and Part 2), it is important to note that, although a clear quantitative reduction in fluorescence was observed in Part 1, the signal remained subjectively detectable across all solutions. On the other hand, in Part 2, greater difficulty in identifying fluorescence was found in MB-dominant dilutions and in the overlay and color map modes, which may reflect the variability of inter-individual tissues. The apparent discrepancy between the results of Parts 1 and 2 may reflect the differences between a simplified in vitro environment and the complex optical and biochemical conditions of biological tissues. In addition, both ICG and MB interact with tissue proteins, particularly albumin, which can modify their fluorescence behavior in situ. In addition, Part 2 relied on a single specimen for each dilution, and individual variability in tissue cadaver composition and protein content may have further influenced fluorescence visualization. Given that the environment variables, imaging system, SPY-PHI application settings, and the distance from the specimens can affect the fluorescence intensity [17,22], in Part 1 and Part 2, all experimental conditions were fully standardized. However, in Part 2, standardization was not fully applicable to the individual variables related to the cadavers that were inevitably present (e.g., skin pigmentation, tissue hydration, fat tissue proportion) and were not accounted for in this study. The authors selected fresh feline cadavers to ensure anatomical similarity; however, certain individual biological variables could not be controlled. It is recognized that albumin can enhance the fluorescence emission of both ICG and MB [19,23], and that ICG naturally binds to tissue proteins and albumin, both in vivo and in cadaveric studies, making pre-mixing ICG with albumin unnecessary in those cases [23,24,25]. The individual variable level of albumin and other proteins in tissues could potentially influence fluorescence visualization and signal intensity. However, there are no data in human or veterinary patients about the relationship between individual variability in binding proteins in tissues and the fluorescence emitted by these tracers during clinical procedures [23]. This hypothesis represents one possible factor to consider in daily clinical practice when interpreting fluorescence imaging and using these technologies, and it should be acknowledged as a potential source of variability in ex vivo or cadaver-based studies.

Three observer groups with three levels of experience were included in Part 2 of this study; although overall differences between observer experience groups were not substantial, moderate- and low-experience observers showed a slightly higher proportion of fluorescence recognition in overlay mode, particularly for the MB-dominant solution. These data suggest that NIRF image interpretation may not require extensive prior experience; however, prospective studies with adequate statistical power are needed to confirm this hypothesis.

The primary limitation of this study is the small sample size of the cadaveric models (Part 2). However, the authors are not aware of any previous studies that have evaluated intermolecular quenching in cadavers; therefore, this should be considered an exploratory study, and the results of inter-solution comparisons should be interpreted with caution. This study did not specifically control for cadaver-related variables such as tissue composition, protein content, and hydration status, and this could be considered a limitation. However, the use of cadaveric models inherently encompasses a wide range of biological variability, closely resembling the heterogeneity encountered in clinical practice. Nonetheless, further clinical studies are warranted to assess the impact of these variables in vivo, considering different tracer solutions, patient-specific conditions, and different SLN mapping scenarios. Additionally, the results of the present study, both in terms of quantitative assessment and image interpretation, are inherently linked to the SPY-PHI imaging system; therefore, their generalizability to other NIRF systems and setups remains uncertain. Future studies should replicate these experiments and directly compare different NIRF technologies. Overall, this exploratory study highlights the need for further physical–chemical investigations and in vivo validation with larger samples to better define patient-specific factors and optimize clinical NIRF lymphography.

## 5. Conclusions

In conclusion, this exploratory study demonstrated a quenching effect of MB on ICG fluorescence in both in vitro experiments and in a feline cadaveric model, as evidenced by quantitative and qualitative fluorescence analysis. Solutions containing the ICG–MB combination showed lower fluorescence intensity compared to the ICG-only solution; however, among the ICG-MB solutions, the ICG-dominant showed a higher fluorescence intensity and a better performance in the lymphography interpretation.

The MB may influence the interpretation of lymphographic NIRF images, via an intermolecular quenching effect, when ICG-MB equal and MB-dominant solutions are used. Hence, careful optimization of the ICG–MB ratio, selecting an ICG-dominant solution, may reduce the risk of clinically relevant quenching while preserving the surgical guidance benefits of MB during removal of SLN.

Future confirmatory studies should include a substantially larger number of specimens to allow appropriate statistical comparisons and to better account for inter-individual variability.

## Figures and Tables

**Table 1:** Proportion of participants on the questionnaire who recognized fluorescence in the lymphography and the popliteal lymph nodes using different solutions and different NIRF modes.

	ICG- Only	ICG- Dominant	ICG-MB Equal	MB- Dominant
**Lymphography**				
**Overlay mode**				
High experience	100%	25%	75%	0%
Moderate experience	100%	0%	50%	0%
Low experience	100%	75%	50%	25%
**Color map mode**				
High experience	100%	0%	0%	0%
Moderate experience	100%	25%	0%	0%
Low experience	100%	25%	0%	0%
**Contrast mode**				
High experience	100%	100%	100%	100%
Moderate experience	100%	100%	100%	100%
Low experience	100%	100%	100%	100%
**Excised popliteal lymph node**				
**Overlay mode**				
High experience	100%	100%	100%	50%
Moderate experience	100%	100%	100%	25%
Low experience	100%	100%	100%	50%
**Color map mode**				
High experience	100%	100%	100%	100%
Moderate experience	100%	100%	100%	100%
Low experience	100%	100%	100%	100%
**Contrast mode**				
High experience	100%	100%	100%	100%
Moderate experience	100%	100%	100%	100%
Low experience	100%	100%	100%	100%

**Table 2:** Median (range) of the fluorescence intensity recorded for the four solutions in the three NIRF modalities.

	ICG- Only	ICG- Dominant	ICG-MB Equal	MB- Dominant
**Lymphography**				
**Overlay mode**				
High experience	3 (2–4)	1 (1–2)	2 (1–2)	1 (1–1)
Moderate experience	2.5 (2–3)	1 (1–1)	1.5 (1–2)	1 (1–1)
Low experience	2.5 (2–3)	2 (1–2)	1.5 (1–2)	1 (1–2)
**Color map mode**				
High experience	2 (2–2)	1 (1–1)	1 (1–1)	1 (1–1)
Moderate experience	2 (2–3)	1 (1–1)	1 (1–1)	1 (1–1)
Low experience	2 (2–2)	1 (1–2)	1 (1–1)	1 (1–1)
**Contrast mode**				
High experience	4 (2–5)	3.5 (2–5)	3.5 (2–5)	2 (2–3)
Moderate experience	3.5 (3–4)	3.5 (3–4)	4 (4–5)	2 (2–3)
Low experience	3 (2–3)	3 (2–3)	4 (3–5)	2 (2–3)
**Excised popliteal lymph node**				
**Overlay mode**				
High experience	4.5 (3–5)	5 (5–5)	4.5 (3–5)	1.5 (1–2)
Moderate experience	4 (4–5)	5 (4–5)	3.5 (3–5)	1 (1–2)
Low experience	4.5 (4–5)	4.5 (4–5)	4.5 (3–5)	1.5 (1–3)
**Color map mode**				
High experience	5 (5–5)	5 (5–5)	4.5 (4–5)	4 (2–5)
Moderate experience	4.5 (4–5)	5 (5–5)	3.5 (3–4)	2 (2–3)
Low experience	4.5 (3–5)	4.5 (3–5)	3 (2–4)	2 (2–4)
**Contrast mode**				
High experience	5 (5–5)	5 (5–5)	4.5 (3–5)	4.5 (2–3)
Moderate experience	5 (5–5)	5 (5–5)	3.5 (3–5)	2 (2–2)
Low experience	5 (5–5)	5 (5–5)	4 (4–5)	2.5 (2–3)

## Data Availability

All data generated or analyzed during this study are included in this article. Additional datasets are available from the corresponding author upon reasonable request.

## Abbreviations

The following abbreviations are used in this manuscript:

MB	Methylene Blue
ICG	Indocyanine Green
NIRF	Near-Infrared Fluorescence
ROI	Region Of Interest
SLN	Sentinel Lymph Node
VTH	Veterinary Teaching Hospital

## Appendix A

### NIRF Lymphography Protocol on Feline Cadavers

Cadavers were stored within 1 h from death at +4 °C and maintained under refrigerated conditions without freezing; the lymphography was planned within 24 h. All cadavers were kept at room temperature for 1 h prior to NIRF lymphography.

Immediately before imaging, the hind limbs were widely clipped, taking care to avoid damaging the skin and, therefore, the superficial lymphatic vessels. Hairs were removed from the digits to the iliac crest and ischiatic tuberosity, including the inguinal region medially. Surgical drapes were used to isolate the limb from the surrounding environment in order to standardize the NIRF lymphography images acquired.

Lymphatic mapping was consistently initiated at the dorsal metatarsal region, defined as the dorsal aspect and delimited proximally by the base of the second and fifth metatarsal bones and distally by the metatarsophalangeal joints [20].

For each lymphography, one of the 4 solutions was used and injected into one of the 4 hind limbs included. Each solution had a total volume of 0.8 mL and was intradermally injected at multiple sites to uniformly cover the whole dorsal metatarsal region. Gentle massage and passive limb mobilization were applied to facilitate lymphatic flow.

Lymphatic drainage pathways were visualized using the NIRF system. If no lymphatic drainage was visualized, or if drainage was observed but appeared interrupted and did not progress further despite 10 min of limb massage and mobilization, the procedure was considered unsuccessful and the limb was excluded. The experiment was then repeated using the same solution on a different, previously unused limb. Following identification of lymphatic vessels and popliteal lymph nodes, the popliteal lymph nodes were excised and analyzed using NIRF imaging. The NIRF images of both lymphography and excised lymph nodes were acquired using all available imaging modalities.

**Table A1:** Mean ± SD or median (range) of the fluorescence intensity recorded for the four solutions in the three NIRF modalities.

	Overlay	Color Map	Contrast
**ICG-only**	101.8 ± 3.3 ^†^	104.0 ± 5.6 ^†^	101.6 ± 4.3 ^#^
**ICG-dominant**	71.2 ± 3.4 ^B^	81.8 ± 3.1 ^A^	36.8 ± 1.9 ^C,^ *
**ICG-MB equal**	30.4 ± 2.7 ^B, ‡^	38.6 ± 1.1 ^A, ‡^	20.0 (20.0–22.0) ^C,^ *
**MB-dominant**	36.0 (36.0–39.0) ^A, ‡^	35.2 ± 2.8 ^A, ‡^	28.6 ± 2.1 ^B,^ *

Statistics: Differences between the three NIRF modalities within each solution: ^A^ > ^B^ > ^C^ for *p* < 0.005; differences between the four ICG solutions within each NIRF modality ^†^ > ^‡^ for *p* < 0.03 and ^#^ > * for *p* < 0.0001.

**Table A2:** Differences between expertise groups across solutions and visualization modes for fluorescence recognizability (Question 2) in lymphographies and excised lymph nodes (Bonferroni *p*-values).

**Lymphographies**			
	**Overlay**	**Color Map**	**Contrast**
ICG-only	0.53	0.37	0.22
ICG-dominant	0.09	0.37	0.33
ICG-MB equal	0.73	0.99	0.60
MB-dominant	0.37	0.99	0.99
**Lymph nodes**			
	**Overlay**	**Color Map**	**Contrast**
ICG-only	0.83	0.26	0.99
ICG-dominant	0.29	0.11	0.99
ICG-MB equal	0.66	0.049 *	0.61
MB-dominant	0.66	0.16	0.25

Note: * statistically significant.
